# Case Report: Primary breast leiomyosarcoma in an 84-year-old male

**DOI:** 10.3389/fonc.2025.1660377

**Published:** 2025-10-02

**Authors:** Jie Yang, Wei Liu, Jie An, Cheng Jiao, Zhi Li, Shuai Qi, Chen Hao, Yao Zhang, Hui-Lin Wang, Jun Guo

**Affiliations:** ^1^ Cadre Ward, Bethune International Peace Hospital, Shijiazhuang, China; ^2^ Department of General Surgery, Bethune International Peace Hospital, Shijiazhuang, China; ^3^ Department of Pathology, Bethune International Peace Hospital, Shijiazhuang, China; ^4^ Department of Radiology, Bethune International Peace Hospital, Shijiazhuang, China

**Keywords:** breast leiomyosarcoma, male, advanced age, indolent growth, senescent microenvironment, case report

## Abstract

Primary breast leiomyosarcoma is an extremely rare malignancy originating from mesenchymal tissue. Fewer than 10 male cases have been reported globally. This paper reports an 84-year-old male patient. This represents the oldest reported case in the current literature. A painless, slowly enlarging mass was present in his right breast. The mass had a 10-year history. This contrasts sharply with the typically rapidly progressive pattern documented in previous literature. Clinical examination revealed a mobile mass measuring 12 cm × 10 cm in the right breast. No lymphadenopathy was detected. Ultrasound showed a hypoechoic lesion classified as BI-RADS 4a. Magnetic resonance imaging demonstrated plateau-type enhancement. The patient underwent simple mastectomy. Axillary lymph node dissection was not performed. Postoperative pathology and immunohistochemistry confirmed the diagnosis of breast leiomyosarcoma. The patient declined adjuvant radiotherapy. Follow-up at 6 months postoperatively showed no local recurrence or metastasis. This case indicates several points to clinicians. Immunohistochemistry serves as the cornerstone for diagnosing spindle cell tumors of the breast. R0 surgical resection constitutes the core approach for achieving cure. Decisions regarding adjuvant therapy require full consideration of host age and tumor biological behavior. The senescent microenvironment in elderly patients may suppress aggressive tumor progression.

## 1 Introduction

Primary breast leiomyosarcoma (PB-LMS) is an exceptionally rare malignant tumor, accounting for approximately 1% of breast tumors and less than 5% of soft tissue sarcomas (1). Its pathogenesis remains unclear but may involve malignant transformation of vascular walls, nipple smooth muscle, or myoepithelial cells (2). It typically exhibits rapid growth and demonstrates highly aggressive biological behavior (3). Occurrence in males is exceedingly rare, with fewer than 10 cases indexed in PubMed databases as of 2024 (2). Reported male cases predominantly affect individuals aged 48–68 years and typically present with rapidly enlarging masses over several months (4). This report describes an 84-year-old Asian male with PB-LMS exhibiting intermediate-grade (G2) histology yet demonstrating an indolent growth course spanning 10 years—a distinct feature from prior reports with significant clinical and biological implications. This case was reported in accordance with the Surgical CAse REport (SCARE) 2023 Guidelines (5).

## 2 Case presentation

### 2.1 Patient information

An 84-year-old male was admitted with a 10-year history of a painless right breast mass. The mass, initially discovered incidentally and approximately date-sized, showed no redness, pain, nipple discharge, or retraction. The patient was advised of a benign lesion during prior outpatient evaluation and received no treatment. The mass exhibited continuous slow growth over a decade. Recent discomfort during movement occurred due to its pendulous nature, prompting surgical consultation. Medical history revealed no gynecomastia, hypertension, diabetes mellitus, or coronary artery disease. The patient had a 60-year smoking history (approximately 20 cigarettes per day) and continued smoking at presentation. Alcohol consumption was denied.

### 2.2 Clinical presentation

**Figure 1:**
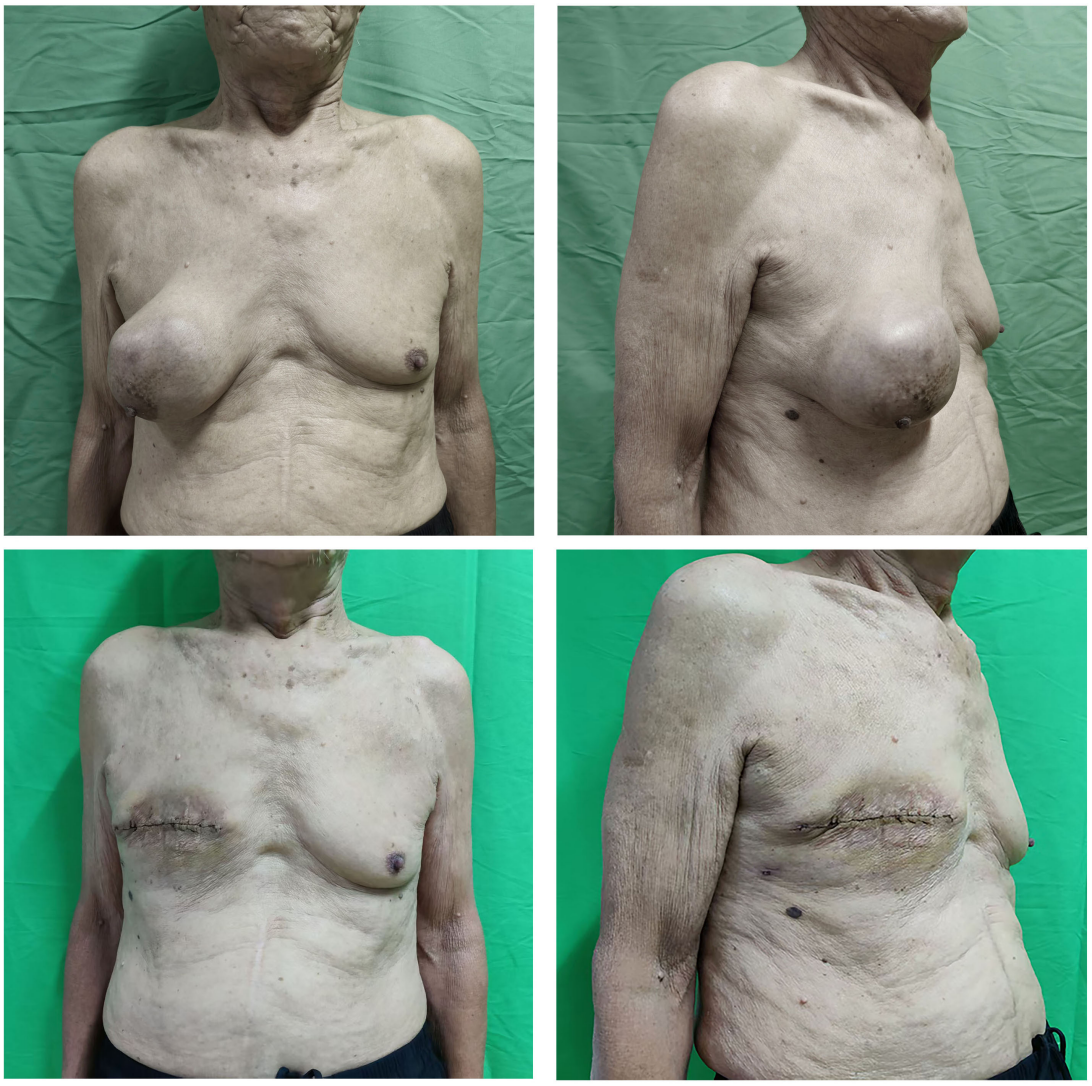
Preoperative and postoperative appearance of the patient.

Physical examination revealed a firm, well-defined 12 cm × 10 cm mass in the right breast with good mobility (**Figure 1**). No palpable lymphadenopathy was detected in bilateral axillary, supraclavicular, or cervical regions.

### 2.3 Timeline

The patient had no history of gynecomastia. No family history of breast cancer or other malignancies was reported.

### 2.4 Diagnostic evaluation

**Figure 2:**
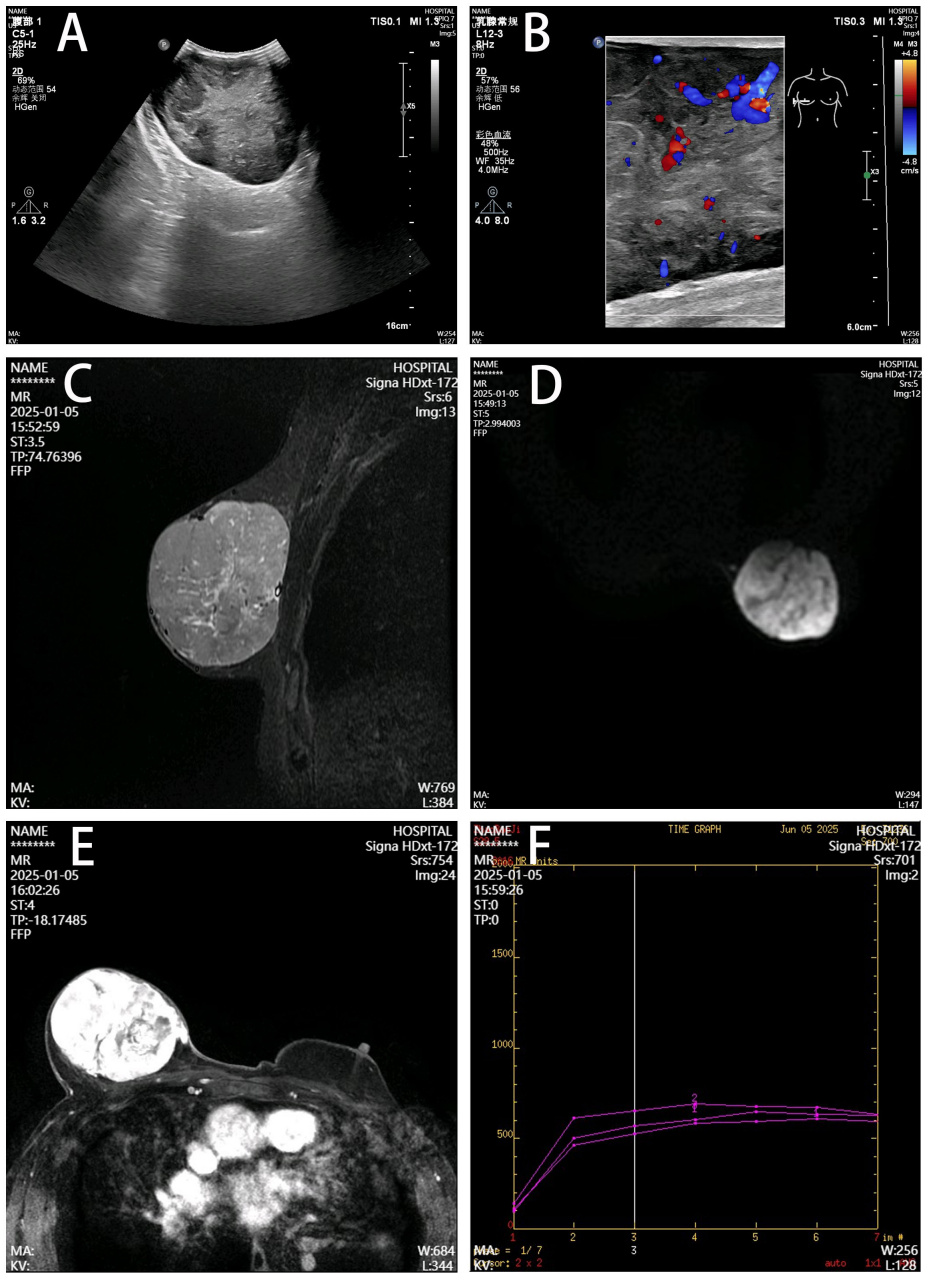
Imaging features of the right breast mass. **(A)** Ultrasonography, Well-defined hypoechoic mass (12.3×9.4×5.5 cm) with heterogeneous echogenicity. **(B)** Color Doppler, Moderately abundant internal blood flow. **(C)** T2 fat-suppressed MRI, Lobulated mass showing heterogeneous hyperintensity (arrow). **(D)** DWI, Hyperintense signal with clear margins. **(E)** Contrast-enhanced MRI, Marked heterogeneous enhancement with capsular discontinuity (arrowhead). **(F)** Time-intensity curve, Rapid initial rise followed by plateau kinetics.

Breast ultrasonography revealed a 123 mm × 94 mm × 55 mm hypoechoic mass with well-defined margins and relatively rich internal blood flow signals in the right breast region, assessed as BI-RADS category 4a. Magnetic resonance imaging (MRI) demonstrated a large right breast mass showing T1 hypointensity and heterogeneous slight hyperintensity on T2 fat-suppressed sequences, measuring 9.2 cm × 7.2 cm × 7.1 cm. Contrast-enhanced scan exhibited marked heterogeneous enhancement with a rapid initial rise followed by plateau-type time-intensity curve (TIC) kinetics (**Figure 2**). Tumor markers (CA153, CA125) were within normal limits. Chest, abdominal, and pelvic computed tomography (CT) showed no evidence of metastasis.

### 2.5 Therapeutic intervention

Preoperative evaluation revealed no surgical contraindications. Ultrasound (BI-RADS 4a) and contrast-enhanced MRI showed a well-circumscribed, plateau-type lesion without spiculation or microcalcifications—features that argued against carcinoma. Although preoperative biopsy remains the standard of care, multidisciplinary discussion concluded that the lesion would most likely be of mesenchymal origin, since core biopsy frequently yields inconclusive results for such spindle-cell lesions (6, 7), and the patient, after informed counseling, opted for direct excision. We therefore omitted percutaneous biopsy and proceeded to simple mastectomy of the right breast under general anesthesia (resection encompassing the tumor and overlying skin, preserving the pectoral fascia). Gross examination showed an encapsulated tumor without adhesion to the pectoral fascia. Intraoperative frozen-section biopsy indicated “malignant mesenchymal tumor with negative margins.” Given the absence of clinically apparent lymphadenopathy, axillary lymph node dissection was not performed. One drainage tube was placed intraoperatively.

### 2.6 Follow-up and outcome

**Figure 3:**
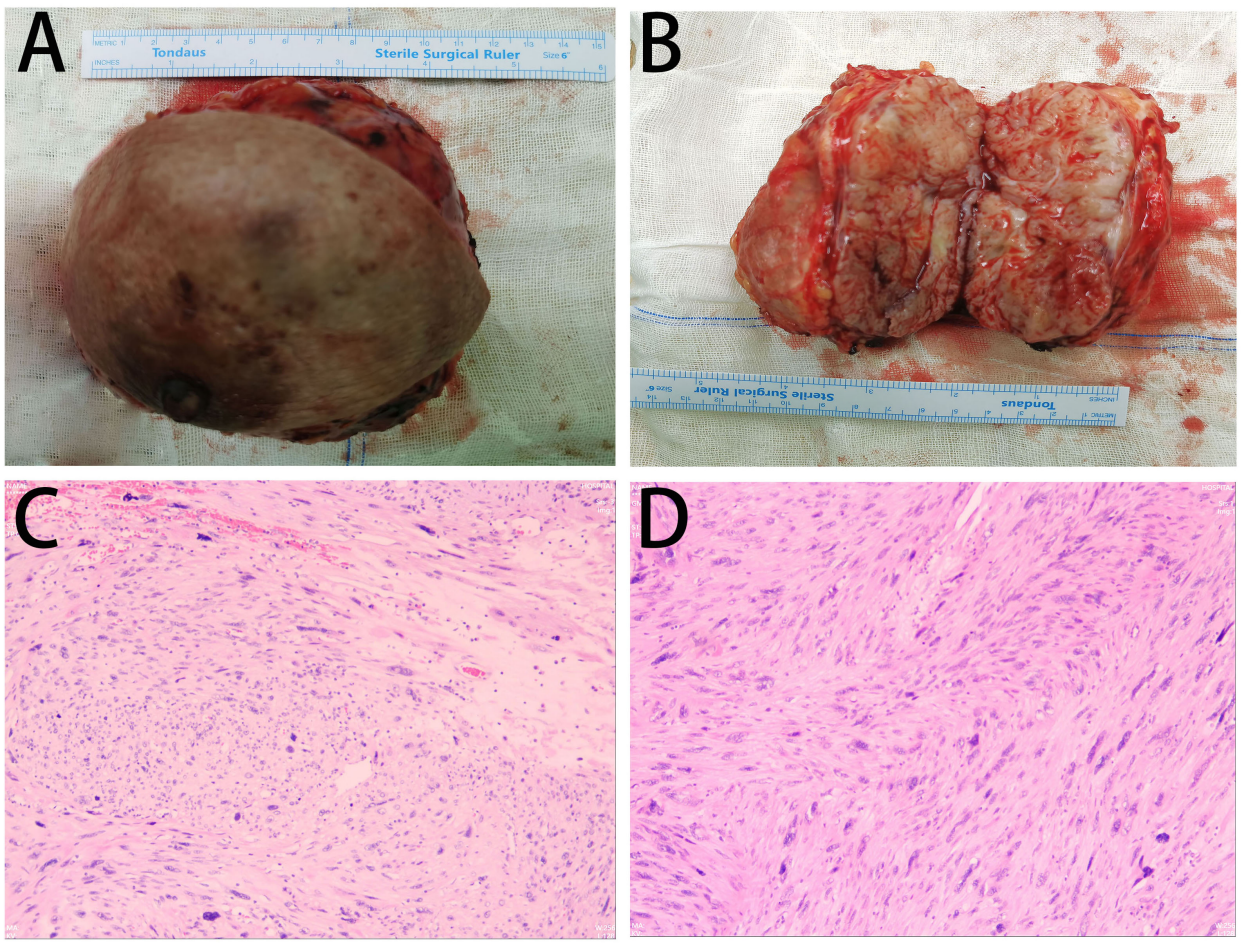
Pathological findings: **(A, B)** Gross specimen of the right breast mass (12 cm × 10 cm) with a grayish cut surface. **(C, D)** Intraoperative frozen section microscopy: Subcutaneous mesenchymal spindle cell tumor (10 cm × 8 cm × 4.5 cm) showing significant cellular pleomorphism, focal hemorrhagic necrosis, readily identifiable nuclear mitotic figures (4–8/10HPF), and atypical nuclear mitotic figures.

**Figure 4:**
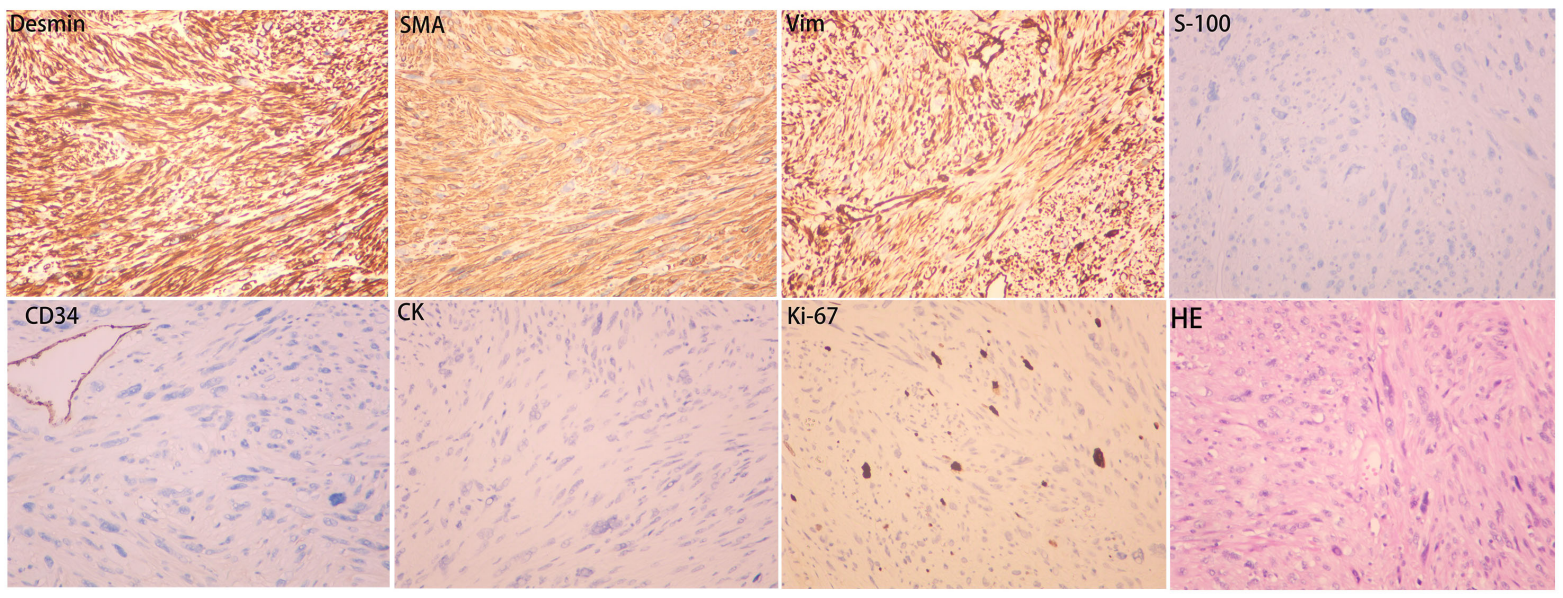
Immunohistochemistry results: SMA (+), Desmin (+), Vim (+), CK (−), S-100 (−), CD34 (−), Ki-67 (+, 15%).

Routine postoperative care was administered. Drainage fluid volume was less than 10 mL over a 24-hour period on postoperative day 5. The drainage tube was removed. The patient was discharged. No adverse events were reported during the treatment period. The patient expressed high satisfaction with the treatment. Gross pathological examination identified a subcutaneous mesenchymal spindle cell tumor measuring 10 cm × 8 cm × 4.5 cm, exhibiting significant cellular pleomorphism, focal hemorrhagic necrosis, readily identifiable nuclear mitotic figures (4–8/10HPF), and atypical nuclear mitotic figures (**Figure 3**). IHC results were as follows: CK (−), Vim (+), CK5 (−), CK7 (−), CK19 (−), SMA (+), Desmin (+), S-100 (−), CD34 (−), Ki-67 (+, 15%) (**Figure 4**). The diagnosis of PB-LMS was confirmed. Adjuvant radiotherapy was recommended but declined by the patient due to financial constraints. No local recurrence or distant metastasis was detected at the 6-month postoperative follow-up.

## 3 Discussion

PB-LMS is an exceptionally rare malignancy of mesenchymal origin, with fewer than 100 cases reported in the literature, and female incidence is significantly higher than male incidence (1, 8, 9). Previous literature demonstrates that male PB-LMS typically exhibits aggressive biological behavior with rapid growth over short timeframes. As reported by Cheikh TE, et al (2), a 65-year-old patient presented with a disease course of 3 months. Other documented cases generally manifested clinical symptoms requiring medical attention within months to 3 years after onset. (see **Table 1**). In this case, the patient’s right breast mass demonstrated slow growth over 10 years, reaching a diameter of 12 cm without pectoral fascia invasion or distant metastasis. Histological features and clinical progression showed inconsistency.

**Table 1:** Clinically reported features of male breast leiomyosarcoma.

No	Author/s (year of publication)	Age (years)	Size (cm)	Duration of disease (months)	Treatment	Outcome and follow-up
1	Crocker and Murad (1969, cited in Fujita et al., 2011) (14)	51	5	NA	RM	NA
2	Hernandez FJ (1978) (15)	53	4x3x2	12	MRM	Alive,14 months
3	E Farkas (1991) (16)	61	NA	NA	NA	NA
4	Alessi and Sala (1992) (17)	62	NA	24	NA	Alive,72 months
5	Boehm D, et al. (2010) (18)	62	4.4x3.4x3.4	3	MRM	Alive,24 months
6	Masannat Y,et at (2010^)^ (19)	59	2	NA	SM	Alive,26 months
7	Arsalane A, et al. (2017) (20)	68	8x9	12	RM +ALND	Alive,24 months
8	Villegas JB,et at (2018) (4)	48	9.0x8.0	36	SM	Alive,1 months
9	Ely Cheikh T, et al (2021) (2)	65	8.7x8.5x7.5 2.8x1.8x1.5	3	Left WLE, +Right SM	Alive,11 months

Cm, centimeter; WLE, wide local excision; RM, radical mastectomy; SM, simple mastectomy; MRM, modified radical mastectomy; NA, Data records in some early literature were incomplete. Some information was missing.

The indolent phenotype in this case suggests that the senescent microenvironment in advanced age may inhibit tumor progression. On one hand, the aging-associated immunosuppressive microenvironment weakens immune surveillance. In elderly individuals, reduced diversity of the T-cell receptor repertoire occurs. T-cell exhaustion (high expression of programmed cell death protein 1 [PD-1] and cytotoxic T-lymphocyte-associated protein 4 [CTLA-4]) and decreased cytotoxicity of natural killer (NK) cells are observed. Concurrently, myeloid-derived suppressor cells (MDSCs) and regulatory T cells (Tregs) accumulate in the tumor microenvironment (TME), leading to weakened immune surveillance (10, 11), This immunosuppressive state may permit long-term tumor cell survival.

On the other hand, reduced angiogenic factors (e.g., vascular endothelial growth factor [VEGF]) and stiffening of the extracellular matrix (ECM) (e.g., excessive collagen deposition) in the senescent microenvironment may physically restrict local tumor invasion and metastasis (10). Additionally, in classic senescence models, factors such as matrix metalloproteinases (MMPs) and interleukin-6 (IL-6) promote invasion. However, certain senescence subtypes (e.g., mitochondrial dysfunction-associated senescence) may reduce pro-inflammatory factor secretion (e.g., interleukin-1α [IL-1α]). Tumor-suppressive factors such as insulin-like growth factor binding protein 7 (IGFBP7) are increased (12, 13). Consequently, the senescence-associated secretory phenotype (SASP) in the aged microenvironment may skew toward a tumor-suppressive phenotype.

In this case, weakened immune surveillance may allow long-term tumor survival. However, ECM stiffening and the tumor-suppressive SASP tendency restrict invasive potential, ultimately manifesting as indolent local growth. This “survival-permissive, invasion-restrictive” microenvironmental balance represents a hypothetical mechanism. It provides new insights into the dissociation between histological grade and clinical behavior in elderly patients. Validation in additional cases and experimental models is required.

The imaging characteristics and 10-year disease course in this case could easily be misinterpreted as a benign tumor, underscoring the heterogeneity and unpredictability of PB-LMS clinical presentations. This case highlights that even intermediate-grade (G2) PB-LMS may exhibit indolent clinical progression, with imaging features mimicking benign lesions. Definitive diagnosis requires histopathological and IHC confirmation. The IHC profile in this case (SMA+, Desmin+, Vim+; negative for epithelial and neural markers) is the gold standard for confirming LMS and excluding other spindle cell tumors, such as metaplastic carcinoma, malignant phyllodes tumor, or angiosarcoma (6).

Radical surgical resection (R0 resection) is the only potentially curative approach for localized PB-LMS. This case achieved R0 resection of the tumor, Axillary lymph node dissection (ALND) was not conducted. Extensive studies demonstrate that breast sarcomas (including LMS) primarily metastasize hematogenously (commonly to lung and bone), with extremely low regional lymph node metastasis rates (7, 21, 22). In a large soft tissue sarcoma (STS) study (n=1,772), the lymph node metastasis rate was 2.6%. A Surveillance, Epidemiology, and End Results (SEER) database analysis of breast sarcomas (n=333) revealed that among 129 patients undergoing ALND, only 6 (4.7%) had axillary lymph node metastasis (23, 24). Eloi Ramelli et al. (2022) reported no significant difference in overall recurrence rates based on initial ALND performance (26% *vs* 41%; OR = 1.11, P = 0.29), suggesting prophylactic ALND may lead to overtreatment in breast sarcoma (25). Therefore, for breast sarcomas (including LMS) without preoperative imaging or intraoperative evidence of lymph node involvement, ALND is not mandatory. The surgical strategy in this case aligns with current evidence-based principles to avoid overtreatment.

Wide local excision was not attempted: with a 12-cm tumor and the minimal breast parenchyma typical of a male patient, this approach was deemed infeasible. Obtaining adequate (>1 cm) margins via WLE would have produced a cosmetically unacceptable deformity and carried a higher risk of positive margins. Simple mastectomy achieved complete tumor clearance with minimal morbidity and is consistent with current sarcoma guidelines (26).

The necessity of adjuvant radiotherapy or chemotherapy for R0-resected tumors lacking high-risk features remains controversial, with limited high-level evidence specific to breast LMS (27). In this case, intermediate-grade (G2) histology, negative margins, absence of lymphovascular invasion, Ki-67 index (15%), and 10-year indolent course suggested low aggressiveness. The potential benefit of adjuvant chemotherapy was highly uncertain (8, 28).thus, chemotherapy was omitted. Current European Society for Medical Oncology (ESMO) guidelines recommend radiotherapy as part of standard treatment for intermediate- to high-grade (G2–G3) LMS (26); regrettably, radiotherapy was declined by the patient. Future studies should identify molecular biomarkers to more accurately predict recurrence and metastasis risk in PB-LMS, thereby guiding adjuvant therapy decisions.

Despite the notably indolent features in this case, the documented risk of delayed metastasis in PB-LMS (occurring up to 15–20 years post-diagnosis) (29, 30) necessitates ongoing vigilance. Long-term (>10 years) vigilant surveillance remains crucial.

This study is a single case report. Validation of the mechanism underlying “senescent microenvironment inhibiting tumor invasion” requires additional cases and animal experiments.

## 4 Conclusion

This report details an 84-year-old male with PB-LMS exhibiting a 10-year indolent course despite intermediate-grade histology (G2). This phenomenon challenges the traditional paradigm of PB-LMS as highly aggressive. We propose that the unique microenvironment of elderly hosts—including senescence-associated secretory phenotype (SASP) reprogramming and extracellular matrix (ECM) stiffening—may suppress invasive potential, thereby shaping indolent phenotypes. This offers a potential mechanistic explanation for the dissociation between histological grade and clinical behavior. The case underscores the cornerstone role of IHC in diagnosing breast spindle cell tumors and the limitations of imaging in predicting sarcoma biology. R0 surgical resection remains the foundation for cure, while adjuvant therapy decisions in elderly patients require highly individualized approaches, particularly integrating host age and observed tumor behavior. The management of this case underscores the critical importance of centralized care for such rare sarcomas in specialized referral centers with dedicated pathologists and multidisciplinary teams (MDT) (31). Future research must prioritize identifying age- and indolence-associated molecular biomarkers in PB-LMS to optimize diagnosis, prognostication, and therapeutic strategies.

## Data Availability

The raw data supporting the conclusions of this article will be made available by the authors, without undue reservation.
